# The impact of instruction and response cost on the modulation of response-style in children with ADHD

**DOI:** 10.1186/1744-9081-6-31

**Published:** 2010-06-04

**Authors:** Renate Drechsler, Patrizia Rizzo, Hans-Christoph Steinhausen

**Affiliations:** 1 Department of Child and Adolescent Psychiatry, University of Zurich, Neumuensterallee 9, 8032 Zurich, Switzerland; 2 Aalborg Psychiatric Hospital, Aarhus University Hospital, Mølleparkvej 10, 9000 Aalborg, Denmark; 3 Clinical Child and Adolescent Psychology, University of Basel, Missionsstrasse 60/62, 4055 Basel, Switzerland

## Abstract

**Background:**

The present study investigated the impact of divergent instructions and response cost on strategic cognitive control in children with ADHD.

**Methods:**

Children with ADHD (N = 34), combined subtype, and control children (N = 34) performed a series of self-paced computerized visual search tasks. The tasks varied by verbal instructions: after a baseline task, children were either instructed to work as fast as possible (speed instruction) or as accurately as possible (accuracy instruction). In addition, tasks were performed with and without response cost.

**Results:**

Both groups modulated latencies and errors according to instructions in a comparable way, except for latency in the accuracy - instruction without response cost, where control children showed a larger increase of response time. Response cost did not affect the modulation of response style in children with ADHD to a larger extent than in controls. However, with instructions group differences related to target criteria became clearly more accentuated compared to baseline but disappeared when response cost was added.

**Conclusions:**

Delay aversion theory and motivational or state regulation models may account for different aspects of the results. Modifications related to task presentation, such as the emphasis put on different details in the verbal instruction, may lead to divergent results when comparing performances of children with ADHD and control children on a self-paced task.

## Background

A fast and inaccurate response style is one of the most salient features of ADHD. In recent years, a number of studies have dealt with the question to which extent the impulsive response style characteristic of ADHD can be related to executive function deficits, to deficient arousal mechanisms, to specific motivational factors or to combinations of these features.

Early studies with the Matching Familiar Figures Test (MFFT) [1] provided empirical evidence for a specific type of fast and inaccurate responding. In its original form, the MFFT is a self-paced visual search task where individuals have to detect an identical matching picture among an array of six similar variants. Several explanatory models of ADHD have departed from studies based on this test. A study on the modulation of speed-accuracy in ADHD [2,3] provided support for the cognitive-energetic model of ADHD [4,5]. In a visual search task with externally paced presentation rate, children with ADHD and controls were instructed to focus either on speed, or on accuracy, or on both. The task followed a response cost design punishing an excess in errors or time according to task set. Compared to normal controls or children of the inattentive subtype, children with ADHD combined subtype were less able to modulate their response style and did not speed up when asked to do so. This finding was in contrast to the spontaneous behaviour observed in ADHD and was interpreted as an inability to allocate attentional resources. Ever since, the majority of studies related to the energetic state model have dealt with the difficulty of children with ADHD to cope with under- or overactivation induced by the slow or fast event rate of tasks (see [6]).

Model assumptions for self-paced paradigms are less specific. In a self-paced task a child determines his or her own event-rate: The stimulus will last on the screen until a button is pressed. Van der Meere and coworkers [7] predicted no differences between children with ADHD symptoms and normal controls on a self-paced task in contrast to externally paced-tests, where children with ADHD should respond more slowly compared to controls. This hypothesis of the authors was confirmed by the results of their study. A characteristic speed-accuracy trade-off could rather be attributed to comorbid Oppositional Defiant Disorder (ODD) than to ADHD. However, according to the original energetic state model, one might also assume a dependency of performance on task characteristics [4,8,9]: if tasks are underarousing and boring, children might speed up in order to enhance arousal. On the other hand, if a task is very difficult and effort demanding, children with ADHD are unable to regulate effort accordingly and might start responding prematurely without sufficiently monitoring their answers for accuracy.

Another major theory that departed from studies of the MFFT is the Delay Aversion Theory, which claims that behavioural problems of children with ADHD reflect attempts to avoid or escape delay [10-13]. In a self-paced visual search task, the amount of time which a participant will spend on the visual search and the degree of certitude he needs in order to respond are matters of deliberate choice and depend on individual standards. Accordingly, the Delay Aversion Theory claims that a child with ADHD will always place the reduction of delay above accuracy or reward. Studies with the MFFT and variations showed that children with ADHD responded more quickly and made more errors than controls. When errors were punished by enforced trial length, children with ADHD withheld responses as well as controls but did not benefit from extra time to improve efficiency [14].

According to Executive Function (EF) models of ADHD an impulsive response style is linked to behavioural disinhibition and associated deficits of the executive function system. These difficulties may be more basic in nature, such as deficits of working memory, time processing and impaired visual search processes, or may be related to higher level processing such as the application of top-down strategy, goal setting or performance monitoring ([15-17], see [18]). To which extent the choice of a premature response deadline [19] in ADHD is related to impaired working memory and deficient inner clock mechanisms still needs to be investigated [20]. It has been argued that in visual search tasks the number of errors and not latency is related to underlying competence [10,21], i.e. to executive function (see [22]). Visual search abnormalities in ADHD, on the other hand, seem to be strategic rather than cognitive in nature [13,23]: children with ADHD initiate search later, spend less time on the search, and use less systematic scanning strategies. Studies with normal individuals have related atypical speed-accuracy trade-offs in the MFFT to deficiencies in monitoring and metacognitive control [24,25]. There is evidence hat children with ADHD are impaired in performance monitoring [26] and in self-evaluation (for a review see [27]), as well as in the self-initiated use of appropriate metacognitive strategies, such as semantic clustering in order to facilitate verbal learning. But when explicitly instructed, they make use of these strategies and improve in performance [28,29].

The executive function theory of ADHD has been supported empirically (see [30,31]). However, EF deficits in ADHD are neither universal, nor specific [32,33]. Also, several replication studies with the MFFT failed to discriminate between children with ADHD and controls [34-37]. The theoretical framework is further complicated by the fact that so-called "hot executive functions"[38] comprising a range of functions such as emotional decision-making and learning by reward and punishment are also compromised in a subset of children with ADHD (see [20,39-41]). Motor timing performance and inhibition may improve with reward in children with ADHD [42-45] indicating an association between cognitive and motivational mechanisms. These results are in line with state regulation theory: reward is expected to affect effort and in consequence to improve performance [40]. In contrast, delay aversion theory predicts that children with ADHD prefer smaller-immediate over larger-delayed rewards (choice impulsivity). To date, however, studies on the impact of reward on performance in ADHD are far from being consistent [20,40,46,47]. In addition, the role of comorbid ODD is still unclear. It has been suggested that improvements due to reward are larger in children with ADHD comorbid for ODD than with "pure" ADHD [48], possibly because performance deficits in ODD/conduct disorders (CD) may be rather attributed to motivational than to executive function deficits ([30], see the meta-analysis by [49]). However, other studies found increased inhibitory control deficits [7], or EF-deficits in task-monitoring and planning [50] in ADHD plus ODD, or failed to find differences between ADHD-only and ADHD plus ODD related to the impact of reward [44].

In personality research, speed-accuracy processing has been linked to traits of impulsiveness and reflectivity [51]. Dikman and Meyer [52] showed that normal adults with high self-rated impulsiveness probably rely on more "holistic" processing strategies, which they do not modify even when more time is available. Recurring to Kagan's [1] definition of four response styles, Rosencwaig and Corrover [24] distinguish between fast and accurate individuals, who alternate between holistic and analytic processing strategies according to task demands, reflective individuals with a preference for analytic strategies, impulsive individuals with a preference for holistic processing, and slow but inaccurate individuals who have difficulties in implementing both strategies although they show good metacognitive control. An impulsive response style, therefore, may imply that children do not even try to resolve the task cognitively, but respond randomly [53]. The evaluation of the speed-accuracy trade-off itself has been approached from different methodological standpoints, to begin with classical cognitive psychology over signal detection theory ([54,55], for reviews see [56,57]) and proceeded to methods based on algorithms defining task efficiency [58]. Another possibility is the use of different instructions that induce varying emphasis on accuracy or on speed across tasks (see [57]).

It is the goal of the present study to examine the influence of metacognitive control on performance in ADHS and to determine to which extent children with ADHD are able to modulate the speed-accuracy trade-off by conscious strategic decisions. We will investigate whether children with ADHD modulate their response style on a self-paced visual search task to the same extent as control children when instructed to do so. To this end, performances of children with ADHD will be compared to those of normal control children, first, when choosing a response style spontaneously without any specifying instruction, and secondly, when instructed to put the emphasis alternately on accuracy or speed. Thirdly, the study will investigate the impact of response cost on either instruction on the modulation of performance by alternatively punishing an excess in errors or in latency.

Given the diversity of coexisting and complementary explanatory models, various predictions may be made. According to the delay aversion theory, one would expect children with ADHD to naturally opt for a fast but inaccurate response style, independently from instruction or reward. When explicitly instructed to focus on errors they should not or slow down their response speed less compared to controls. Accelerating response speed, on the other hand, would be in accordance with their spontaneous response style and they should respond faster or at least as fast as control children when asked to speed up. Response cost should not make a difference in terms of latency, because children with ADHD are presumed to be aversive to delay and not to be reward maximizers. Therefore, with or without response cost, they should consistently respond faster than control children when asked to optimize accuracy. According to executive function theory, three different predictions are possible. If the underlying deficit is essentially related to basic cognitive deficits, one would expect fast and inaccurate responding in children with ADHD when no specific instruction is given, due to deficient inhibitory control, inadequate timing and working memory deficits. In this case, group differences especially with regard to the number of errors should remain constant throughout the tasks and children with ADHD should not benefit over-proportionally from response cost compared to normal control children.

If, on the other hand, the problem is mainly located in the self-initiated application of strategy, explicit instructions could help children with ADHD to improve metacognitive control. Again, group differences should be most pronounced when no specific instruction is given. But with the emphasis explicitly put on strategic aspects, children with ADHD might be enabled - at least momentarily - to exert metacognitive control instead of acting impulsively without thinking. In consequence, with explicit instruction performances of children with ADHD and controls should become more similar compared to baseline. This should especially apply to target measures, i.e. the number of errors under accuracy instruction and latency under speed instruction. If, in contrast, deficient metacognitive control is essentially motivational and not cognitive, one would expect performance differences between groups only to diminish in the response cost conditions. In the non-reward conditions, children should mostly stick to or even accentuate their spontaneous response style. These latter hypotheses would also be in line with state regulation predictions on the regulation of effort, which may be influenced by reward.

## Methods

### Participants

34 children with ADHD (combined subtype) and 34 normal controls participated in the study. Children were closely matched for age, IQ and gender (Table 1). Children with ADHD were recruited via services of the Department of Child and Adolescent Psychiatry, University of Zurich, Switzerland, via local child and adolescent psychiatrists in private practice, and via the Swiss association for parents of children with ADHD (Elpos). Controls were recruited from public schools in Zurich and surrounding areas. Children had to meet the following criteria: IQ > 80, age 7-13 years, and no known neurological disease or acquired brain injury. Intelligence (IQ) was measured individually by a short form of the German version of the revised Wechsler Intelligence Scale for children (HAWIK III), including the subtests Block Design, Picture arrangement, Arithmetic, and Vocabulary [59]. All but 3 children had received a diagnosis of ADHD before entering the study. The Conners Teacher Rating Scale CTRS [60] and the German version of the SNAP-IV- Rating Scale [61] were used as first selection instruments.

**Table 1:** Description of the samples

	ADHD (N = 34)	Controls (N = 34)	*p*
Age (mean, SD)	10.2 (2.0)	10.1 (1.82)	n.s.
range	7.1-13.7	7.1-13.9	
IQ (mean, SD)	105.6 (14.4 )	106.5 (15.1)	n.s.
Ratio boys/girls	29/5	28/6	n.s.
**Parent Ratings SNAP **(mean raw scores, SD)			
Inattention	15.9 (5.8)	6.6 (5.1)	.000
Hyperactivity	6.8 (3.4)	1.4 (1.7)	.000
Impulsivity	5.8 (2.8)	2.0 (1.9)	.000
Oppositional/Aggressive	9.3 (5.7)	4.0 (3.1)	.000
**SDQ **(mean raw scores, SD)			
Hyperactivity	7.5 (1.9)	2.5 (2.1)	.000
Behavioral problems	3.5 (2.6)	1.3 (1.0)	.000
**Teacher Ratings CTRS **(mean T-scores, SD)			
ADHD Index	65.9 (7.3)	47.6 (6.3)	.000
DSM IV Inattentive	65.0 (8.0)	47.6 (6.8)	.000
DSM IV Hyperactive- Impulsive	64.0 (9.4)	47.6 (8.0)	.000
Oppositional	59.0 (9.9)	51.1 (7.3)	.000

Parents of children who met the diagnostic criteria of ADHD combined subtype were invited to a diagnostic interview, including a standardized checklist of ADHD symptoms in different settings (the Parental Account of Children's Symptoms Interview (PACS) [62]). Hypescheme, a computerized operational criteria checklist and diagnostic algorithm for *DSM-IV *and *ICD-10 *was used to confirm the diagnosis ([63], for a description of the procedure see [64]). Children who did not meet the Hypescheme criteria for ADHD combined subtype were excluded from the study. Twenty-two out of 34 children with ADHD were taking stimulants but discontinued medication between 48 h to 24 h before testing. Eight children of the ADHD group fulfilled research criteria for comorbid Oppositional Defiant Disorder (ODD) (based on PACS). Control children within the clinical range on the SNAP or CTRS were excluded from the study. Written consent was obtained by all the parents. The study was approved by the Ethical Committee of the Department of Psychiatry, University of Zurich.

### Experimental tasks and procedure

**Figure 1:**
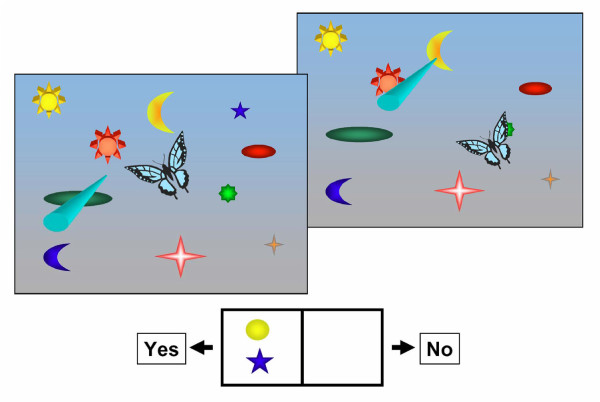
**Items from the visual search tasks**. Target figures remain visible throughout the task. When one of the two targets is detected, children press the "yes"-button, otherwise they press the "no"-button.

A self-paced visual search task presented on computer screen was performed five times, each time with different instruction. Children were seated in front of a 15" computer screen, with one hand placed on a "yes"-, the other on a "no"-button. They had to search for one of two possible target figures, e.g. a blue star or a yellow ball, presented among coloured distractor items (Figure 1). When one of the two targets was detected, children had to press the "yes"-button, otherwise they pressed the "no"-button. As soon as the button was pressed, the next picture appeared. Target figures remained visible throughout the test so that memory load was minimized. Each task consisted of 80 trials with 50% critical and 50% noncritical trials in randomized order. Each test block was preceded by 15 practice trials. Immediate feedback was provided during practice after each slide and at the end of the practice block. In the tests, feedback was provided only at the end of each block of 80 trials. Target figures and distractor items differed between conditions (i.e. between baseline, no reward condition, response cost condition) in order to minimize practice effects and to control for novelty, but all relevant features (size, number, position of targets and distractors) were strictly parallelized. Task difficulty was kept intentionally low, in order to prevent guessing strategies and responding at random. A response cost design was chosen because of its large effects on ADHD as described in the literature [65,66].

Different test instructions were given for each task condition:

1. In the baseline condition, task instructions did not contain any specification concerning speed or accuracy.

2. In the speed - condition (Speed1), children were explicitly told that in this test speed was much more important than accuracy and that they should try to work as fast as possible. At the end of the practice trial they received feedback for both speed and accuracy, but only feedback on speed was explicitly commented on the screen ("Try to respond faster"). When children asked if they could actually ignore accuracy, they where told: "Accuracy is also important, but speed is much more important." This should prevent children from pressing at random. Before starting the test, they were reminded to work as fast as possible.

3. In the instruction for the accuracy - condition (Accuracy1), children where told that accuracy was much more important than speed and that they must avoid errors. After the practice trial, the computer provided feedback for speed and accuracy, but only the accuracy-score was explicitly commented on the screen ("Try to work more accurately"). When children asked if they could ignore speed completely, they were told that latency was also important, but that accuracy was much more important. Before starting the test, they were reminded to work as accurately as possible.

4. In the speed condition with response cost (Speed2-RC), children were again instructed to respond as fast as possible. If the total time used was less than two minutes, they would earn one Swiss Franc (SFR). For every additional second they would lose one cent. Only if more than 15 errors were made, they would be punished by an additional loss of money. When children asked for the amount, they were told that they would lose 10 cents. This additional constraint should prevent children from pressing completely at random. Before starting the test, they were reminded by the program to work as fast as possible in order to maximize earnings.

5. In the accuracy condition with response cost (Accuracy2-RC), children would earn one Franc if no error was made. For each error they would lose 10 cents. They were told that they had plenty of time to respond. Only when time exceeded more than 5 minutes, they would be punished by another loss of money. Again, the amount was not specified. When children asked for the exact amount, they were told that they would lose 10 cents. Before starting the test, they were reminded to work as accurately as possible in order to maximize earnings.

At the end of the tasks, children received feedback about the total time, the number of errors and, in the response cost conditions, the amount of money earned. Immediately after the completion of the response cost tasks, the money was handed out to the children.

In a first session, children started with the baseline condition, followed by other tests. After approximately 30 minutes, Speed1 and Accuracy1 tasks were presented in randomized order. The two response cost tasks (Accurracy2-RC, Speed2-RC) were presented in a second session which took place approximately one week later again in randomized order.

### Statistical analyses

Two separate models were run. In a first step, a three-way repeated measures MANOVA with Group (ADHD vs. controls) by Instruction (speed vs. accuracy) by Reward (no response cost vs. with response cost) was performed, including post hoc tests. In a second step, two ANOVAS were carried out, one for errors and one for median RT, with Group (ADHD vs. controls) as between subject factor and 5 tasks (Baseline, Speed1, Accuracy1, Speed2-RC, Accuracy2-RC) as within subject factor, in order to include baseline performance. In addition, planned comparisons were conducted by calculating contrasts (simple) between baseline (= reference level) and the other tasks. Before conducting the analyses, data were explored for violations of assumptions. To correct for skewed distributions, outliers of median RT were recoded to 2 SD above or below group means. Median RT (MD-RT) was used here instead of mean RT because it is less dependent on RT extremes and outliers that are often observed in children with ADHD. Error measures were log-transformed after a constant had been added. Post-hoc tests were performed by t-tests. Correlations between median RT and the number of errors as a measure of speed-accuracy trade-off were calculated separately for each group (Pearson) and additionally controlled for ODD (partial correlation controlled for SNAP ODD subscale raw scores). To check for the influence of comorbidity, an additional MANCOVA and ANCOVAs controlling for ODD were performed. Earnings from the response cost tasks were compared by t-tests.

## Results

### Impact of instruction versus response cost

**Figure 2:**
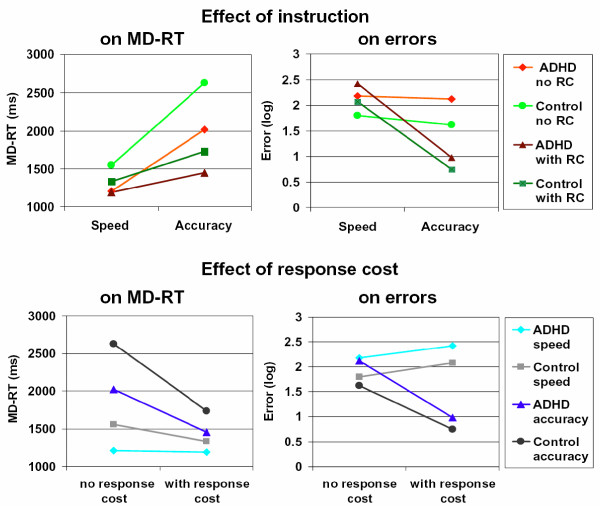
**Effects of instruction and response cost on median RT and errors in four task conditions**.

The three-way repeated measures MANOVA of Group (ADHD/controls) by Instruction (speed/accuracy) by Reward (no response cost/with response cost) (Table 2) revealed significant main effects for Group (p < .001), Instruction (p < 0.001), and Reward (p < .001). Significant interaction effects were found for Instruction by Group (p = .011), Reward by Group (p = .045), and Instruction by Reward (p < .001). The three-way interaction of Group by Instruction by Reward was not significant (p = .250). Univariate and between-subject tests showed that the main effects for Group, Instruction and Reward were highly significant for errors as well as for response time (Table 2), indicating substantial differences between the groups in both measures and an influence of Reward and Instruction on performance. The significant interactions of Instruction by Reward indicated that the number of errors and median RT were both differentially influenced by combinations of Instruction and Reward. Both interactions by Group, i.e. Group by Instruction and Group by Reward, showed significant effects on response speed but not on errors (Group by Instruction: median RT p = .003, error p = .085; Group by Reward: median RT p = .027, error: p = .501), although the interaction of Group by Instruction on errors was significant by trend. These findings indicate that the variation of Reward and Instruction differentially affected response speed in children with ADHD and controls. In contrast, it had similar effects on the number of errors in both groups (Table 2, Figure 2).

**Table 2:** Results of MANOVA and univariate tests analyzing the impact of instruction vs. response cost on four task conditions (Speed1, Acurracy1, Speed2-RC, Accuracy2-RC) in children with ADHD (N = 34) and controls (N = 34)

*MANOVA*							
Source		η^2^	*p*	*ANOVAs*	F	η^2^	*p*
**Group**	Wilks' λ = .695 F_(2/65) _= 14.262	.305	**.000**	**Median RT** **Error**	17.260 13.083	.207 .165	**.000** **.000**
**Instruction**	Wilks' λ = .188 F_(2/65) _= 140.281	.812	**.000**	**Median RT** **Error**	98.542 91.797	.599 .582	**.000** **.000**
**Reward**	Wilks' λ = .168 F_(2/65) _= 161.087	.832	**.000**	**Median RT** **Error**	209.675 193.209	.761 .745	**.000** **.000**
**Group by Instruction**	Wilks'λ.= .870 F_(2/65) _= 4.845	.130	**.011**	**Median RT** **Error**	9.222 3.057	.123 .044	**.003** .085
**Group by Reward**	Wilks' λ = .909 F_(2/65)) _= 3.263	.091	**.045**	**Median RT** **Error**	5.102 .458	.072 .007	**.027** .501
**Instruction by Reward**	Wilks' λ = .151 F_(2/65) _= 182.786	.849	**.000**	**Median RT** **Error**	57.004 248.020	.463 .790	**.000** **.000**
**Group by Instruction by Reward**	Wilks' λ = .58 F_(2/65) _= 1.417	.042	*.250*	**Median RT** **Error**	.563 2.707	.008 .039	.456 .105

η^2 ^= partial eta squared

### Comparisons of tasks including baseline

The ANOVA for median RT over five tasks resulted in significant effects for Group (F = 13.199, part. η^2 ^= .167, p = .001), Task (F = 103.270, part. η^2 ^= .610, p < .001), and a significant interaction of Tasks by Group (F = 4.122, part. η^2 ^= .059, p = .006). Post hoc tests revealed that children with ADHD responded faster than control children in three tasks: Speed1, Accuracy1, and in the Accuracy2-RC task (Table 3). Group differences in response speed at Baseline and in the Speed2-RC condition were not significant, although they showed a trend. The ANOVA for errors showed significant effects for Group (F = 14.159, part. η^2 ^= .177, p < .001) and Task (F = 71.450, part. η^2 ^= .520, p < .001), but the interaction of Group by Task was not significant (F = .643, part. η^2 ^= .010, p = .617). Post hoc tests indicated that children with ADHD made significantly more errors than controls in all conditions except for the Accuracy2-RC task (Table 3).

**Table 3:** Median RT and errors (log) in five task conditions in children with ADHD (N = 34) and controls (N = 34)

	Error log (mean, SD)		p	Median RT (ms) (mean, SD)		p
***Condition***	***ADHD***	***Controls***		***ADHD***	***Controls***	

Baseline	1.92 (.57)	1.66 (.39)	**0.034**	1750 (658)	2015 (490)	0.061
Speed1	2.19 (.76)	1.79 (.48)	**0.011**	1210 (384)	1555 (323)	**0.000**
Accuracy1	2.15 (.69)	1.67 (.49)	**0.001**	2021 (706)	2625 (654)	**0.000**
Speed2-RC	2.43 (.64)	2.06 (.61)	**0.018**	1190 (397)	1336 (284)	0.086
Accuracy2-RC	.99 (.70)	.74 (.12)	0.123	1450 (375)	1732 (365)	**0.002**

Analyses of contrasts between Baseline (BL) and the other tasks showed that response speed at Baseline significantly differed from every other condition (BL/Speed1 p < .001; BL/Accuracy1 p < .001; BL/Speed2-RC p < .001; BL/Accuracy2-RC p < .001). There was only one significant interaction effect for MD-RT by Group (p = .007), indicating a more substantial slowing down in control children from Baseline to the Accuracy1 condition than in children with ADHD. Analyses of contrasts with regard to errors showed that the number of errors significantly increased in both groups from Baseline to Speed1 (p = 0.026) and to Speed2-RC (p < .001), decreased from Baseline to Accuracy2-RC (p < .001), and remained constant from Baseline compared to Accuracy1 (p = .170) (see Table 3).

### Impact of ODD symptoms

The exploratory analyses with the SNAP ODD score as covariate did not yield significant effects for ODD: in the repeated measures MANCOVA (group by Reward by Instruction) neither a significant main effect for ODD (p = .224), nor interaction effects of Reward by ODD (p = .553) or Instruction by ODD (p = .544) emerged. Likewise, in the two ANCOVAs (Group by 5 Tasks) effects for ODD (ANCOVA MD-RT: p = .225; ANCOVA error: p = .260) or ODD by Task (ANCOVA MD-RT: p = .992; ANCOVA error p = .628) were not significant.

### Speed-accuracy trade-off

A significant correlation between errors and median RT reflecting a speed-accuracy trade-off was found in the ADHD group for both speed tasks (Speed1: r = -.366, p = .033; Speed2-RC: r = -.460, p = .006), but not for the other conditions (Baseline: r = -.096, p = .589; Accuracy1: r = -.180, p = .309; Accuracy2-RC: r = .299, p = .085). When controlled for ODD, the correlation of the Speed1 task became reduced to a trend (r = -.314, p = .071), whereas the correlation between median RT and errors in the Speed2-RC task slightly increased (r = -.511, p = .002). These differences between correlations with and without control for ODD were statistically not significant (p = .817, p = .793). In the control group, no significant correlation between speed and accuracy emerged (Baseline: r = .013, p = .941; Speed1: r = .045, p = .800; Accuracy1: r = -.202, p = .253; Speed2-RC: r = -.062, p = .734; Accuracy2-RC: r = -.237, p = .178).

### Earnings

The amount of money earned in both response cost tasks did not differentiate between children with ADHD and controls, although in the accuracy condition there was a trend for control children to earn more money (Accuracy2-RC: ADHD = 0.75 SFR, controls = 0.85 SFR, p = .057; Speed2-RC: ADHD = 0.86 SFR, controls = 0.89 SFR, p = .441).

## Discussion

This study explored the effect of different verbal instructions and of response cost on the modulation of speed and accuracy in children with ADHD and normal controls in a self-paced visual search task. As a general result, children with ADHD and control children showed similar effects when modulating their response style with regard to errors. However, they showed different effects with regard to response speed. This general result is in line with findings that accuracy but not latency is related to underlying competence [10,21] and, therefore, is not differentially affected by contextual factors. In the following, the results of the present study will be related to the initial hypotheses and discussed one by one.

### Effects of instruction

When instructed to work as fast as possible, children with ADHD and controls both increased response speed to the same extent from Baseline to Speed1 and made more errors than at Baseline. When instructed to focus on accuracy, i.e. from Baseline to Accuracy1, both groups slowed down response speed, but control children significantly more than children with ADHD. These results are perfectly consistent with the Delay Aversion Theory [6,10-13]. The number of errors, in contrast, did not decrease substantially in the Accuracy1 task compared to Baseline in either group, which was unexpected. Similar effects have been described by the Delay Aversion Theory for children with ADHD who do not improve in accuracy when more time is available [13]. In the present case, however, this effect was observed in both groups. Several explanations may account for this finding. Possibly, there was a general drop of interest because the same type of task was repeated several times during the test session. Also, compared to the baseline task, children had to search for different target figures, which might have caused interference and, thus, led to an increase of errors. In addition, in the control group the explicit focus on metacognitive strategy might have produced a counterproductive effect: At Baseline, after having completed the practice trial with immediate feedback, many control children already worked quite efficiently. When in the Accuracy1 task they were instructed to focus even more on accuracy, consequently control children spent more time on visual search, to be sure that they had not overlooked a target. This strategy did not lead to a noteworthy decrease of errors, so that their response style became inefficient. Obviously, when explicitly instructed to focus on accuracy, control children recur to metacognitive knowledge implying that slowing down response speed should increase accuracy, even when in a particular task this might not be the case. Thus, compared to children with ADHD, control children to a larger extent relied on metacognitive strategy, when instructed to do so. These results differ from predictions that explicit instruction should improve the application of strategy, but provide some evidence for the insufficient use of metacognitive strategy in ADHD.

### Effects of response cost

Response cost had significant effects on both response speed and the number of errors. The effect on errors was similar in both groups: with response cost, all children made more errors in the Speed2-RC task and fewer errors in the Accuracy2-RC task compared to the equivalent tasks without reward. The effect on speed was more complex. The interaction effect of response cost by group indicated that one group was more responsive to response cost than the other. According to the motivational hypotheses - and in contrast to Delay Aversion Theory which predicts no differential influence of reward - , one might have expected a more extensive slowing down of response speed with response cost in children with ADHD. However, this was not the case here and the overall pattern of change differed from predictions: unexpectedly, both groups responded faster in the Accuracy2-RC task compared to Accuracy1, and this unexpected decrease of latency from Accuracy1 to Accuracy2-RC was more important in control children than in children with ADHD. In addition, in the speed condition response cost had no effect on latency in children with ADHD who remained at the same fast pace, whereas control children tended to respond faster. Therefore, response cost seemed to have stronger effects on the modulation of response style in control children than on children with ADHD. Several explanations may account for this counterintuitive finding: The unexpected decrease of reaction time in the Accuracy2-RC task may be attributed in part to practice effects and previous experience. Some children probably remembered that in this type of task slowing down response speed excessively would not result in the desired improvement in accuracy. Therefore, at the second accuracy-instruction they may have opted for an efficient rather than a particularly slow response style. Another explanation lies in the more explicit limitations of time. In Accuracy2-RC an exaggerate time-on-task would be punished by an additional loss of money. Thus, the message that time-on task was also important, although in the first place children should avoid errors, was probably received more clearly in the response cost condition.

The fact that children with ADHD obviously failed to speed up with response cost when explicitly asked to do so, may be explained by their fast response time at Speed1. When encouraged to work as fast as possible in the non-reward condition, children with ADHD obviously reached their upper speed limit. Consequently, even with the prospective of reward they were unable to speed up any further. In contrast, control children only reluctantly adopted a superficial but fast response style, a type of behaviour that is usually penalized at school. Thus, response cost made instructions cognitively more salient: with the prospective of reward and punishment normal children fully realized that an unusual strategy was required.

Given the fact that the modulation of response time with response cost was obviously confounded with other factors, it is difficult to draw conclusion with regard to the initial hypotheses. Taken all together, the results seem related to differences in the metacognitive use of instructions in children with ADHD and controls rather than to differential effects of response cost.

### Differences of performance between children with ADHD and control children in the five task conditions

When performances of five task conditions were directly compared, the following pattern of performance emerged: At Baseline, group differences were small or absent. In the conditions without response cost, i.e. Speed1 and Accuracy1, differences in target measures between children with ADHD and controls were clearly accentuated compared to Baseline: children with ADHD made significantly more errors than controls in the Accuracy1 condition, and they responded significantly faster than controls in the Speed1 condition. With response cost, group differences in these same target measures disappeared: In the Accuracy2-RC task, errors did not differentiate between the groups, and in the Speed2-RC task group differences in respect of latency were no longer significant. Thus, when considering patterns of performance differences, motivational and state regulation theories are supported that predict no or only minor group differences without specific instruction and enhancement of metacognitive control with reward. In the non-reward conditions, children accentuate their spontaneous response style: control children become overly controlled when asked to focus on accuracy, and children with ADHD become excessively fast, when asked to focus on speed. Response cost reduces group differences in target measures, although differences in non-target measures remain.

### Speed-accuracy trade-off and ODD symptoms

As reported in the literature before [67], a speed-accuracy trade-off as indicated by a significant correlation between errors and median RT was not observed in controls. Normal children obviously do not trade in errors for speed, even when encouraged to do so. In children with ADHD only the speed-instructions produced a significant correlation between errors and latencies. Only when explicitly instructed, but not spontaneously at baseline, children with ADHD traded in errors for speed, which has been interpreted as a sign of increased impulsivity [1,24]. Obviously, with some encouragement, children with ADHD are more easily inclined to loosen control. In the present sample, and in contrast to the results by van der Meere and coworkers [7], the presence of a speed-accuracy trade-off was not directly related to the severity of ODD symptoms. In addition, ODD-symptom severity did not affect response-style modulation in any significant way.

### Limitations

First, position effects are confounded in this design to a certain degree with the effects of the response cost conditions. However, a true randomization of task presentation would have led to the withdrawal of reward in a number of cases. In order to avoid negative effects on motivation, we opted for a controlled sequence of conditions. Secondly, no Bonferroni correction for multiple testing was applied to post hoc t-tests. One reason is that the log-transformation of error measures already constitutes a correction that reduces the significance level. In addition, when comparing performance profiles, the accumulation of the beta-error appears as undesirable as the accumulation of alpha-error (see [68]). Furthermore, because the analyses were hypothesis-driven, correction for multiple testing may be neglected to a greater extent. In any case, the application of a correction for multiple testing would not have changed the interpretation fundamentally. With Bonferroni adjustment, a significant difference between groups in the number of errors would remain solely in the Accuracy1 task. Group differences in median RT would remain significant in Speed1, Accurancy1 and Accuracy2-RC (see Table 3). Therefore, one may still conclude that group differences in target measures were accentuated with instruction and tended to disappear when response cost was added.

## Conclusions

Taken all findings together, different aspects of the present results are best explained by different models. When considering patterns of change, the Delay Aversion Theory provided the best prediction: Control children increased response time significantly more than children with ADHD when instructed to focus on accuracy. Response cost did not have a stronger effect on latency in children with ADHD than in control children. Neither instruction nor response cost had differential effects on the number of errors in the performance of children with ADHD and controls. However, when considering differences in performance measures between groups across tasks, results may be better explained by motivational models and state regulation theory. At baseline, children with ADHD only marginally differed from controls with regard to latency. With instruction, group differences in target measures became more accentuated. With response cost, group differences in target measures disappeared. These results provide evidence that modifications related to task presentation, such as the emphasis put on different details in the verbal instruction, the presence or absence of reward, or repetition, may lead to divergent results when comparing performances of children with ADHD and control children on a self-paced task.

## Competing interests

The authors declare that they have no competing interests.

## Authors' contributions

RD developed the design of the study, performed the statistical analysis and wrote the manuscript. PR recruited and tested the participants and participated in the data analysis. HCS participated in the design of the study, reviewed the statistical analysis and reviewed the manuscript. All authors read and approved the final manuscript.
